# Marital Status Is Associated With Treatment Attainment in Pancreatic Adenocarcinoma

**DOI:** 10.1002/jso.70258

**Published:** 2026-04-08

**Authors:** Pamela Chopra Beniwal, Mohammad Saad Farooq, Gracia Maria Vargas, Russell A. Simons, Mark S. Etherington, John T. Miura, Giorgos C. Karakousis

**Affiliations:** ^1^ Perelman School of Medicine University of Pennsylvania Philadelphia Pennsylvania USA; ^2^ Division of Endocrine and Oncologic Surgery Hospital of the University of Pennsylvania Philadelphia Pennsylvania USA

**Keywords:** marital status, marriage, pancreatic adenocarcinoma, PDAC, SEER, surgery, widowed

## Abstract

**Background:**

In patients with pancreatic adenocarcinoma, for whom complex pre‐ and postoperative therapy is necessary, the effects of social support by means of marital status have not been well studied. We therefore sought to assess the relationship of marital status with treatment attainment in patients with pancreatic adenocarcinoma.

**Methods:**

This retrospective national cohort analysis used the SEER database and included adult patients with stage I–III pancreatic adenocarcinoma from 2018 to 2022 (*n* = 24 540). Rates of treatment (surgery, chemotherapy, radiation), time to treatment, and treatment delay (> 6 weeks from diagnosis) were compared between married, divorced, single, and widowed patients.

**Results:**

Of the eligible 24 540 patients, 14 280 (58.2%) were married, 2731 (9.7%) were divorced, 3665 (14.9%) were single, and 3864 (15.7%) were widowed. Multivariable analysis demonstrated decreased likelihood of undergoing surgery for divorced (aOR: 0.61, *p* < 0.001), single (aOR: 0.60, *p* < 0.001), and widowed (aOR: 0.37, *p* < 0.001) patients versus married patients. Likelihood of treatment delay was higher for divorced (aOR: 1.36, *p* < 0.001), single (aOR: 1.43, *p* < 0.001), and widowed (aOR: 1.46, *p* < 0.001) patients versus married patients as well.

**Conclusions:**

Married patients with pancreatic adenocarcinoma had higher rates of surgery and reduced likelihood of treatment delay compared to divorced, single, and widowed patients.

## 1 Introduction

Despite making up only 3.3% of new cancer diagnoses per year, pancreatic adenocarcinoma (PDAC) is responsible for 8.4% of cancer‐related deaths [1]. Additionally, the incidence of PDAC is rising in younger generations [2], and by 2030, PDAC is projected to be the second leading cause of cancer‐related deaths in the United States and the third leading cause of cancer deaths in Europe [3, 4, 5].

Given this, efforts at maximizing initiation and completion of multimodality therapy, which includes surgery, radiation, and systemic therapy, are essential to improve outcomes [6, 7]. Specifically, the decision to have surgery when it is recommended is associated with improved patient outcomes [8, 9]. In addition to this, delays in time to treatment (TTT) from diagnosis for multiple cancers have been shown to be associated with worse patient outcomes [10, 11, 12, 13], and the few studies that have addressed TTT in PDAC have shown that initiating treatment within 6–8 weeks of diagnosis is associated with improved overall survival [14, 15].

An intuitive but understudied aspect of comprehensive care for cancer patients is the role of social support. Patients who have more developed/robust social support systems exhibit better clinical outcomes across multiple types of cancer [16, 17, 18, 19, 20, 21]. The improvement in patient outcomes may be attributed to a variety of factors, including increases in frequency of screenings, transportation options, financial support, access to medical information, cessation of high‐risk behaviors, psychosocial support, and motivation to initiate and adhere to treatment [22, 23, 24, 25, 26, 27].

Marital status, both a prominent form of social support itself and a proxy for stronger social support networks, has a significant impact on survival in cancer patients, but its effect on initiation of therapy and TTT in PDAC has not been comprehensively reported [18, 28]. Coinciding with this, a worrisome trend is the increased prevalence of social isolation over the past decade, with older adults and minoritized populations being particularly vulnerable [29, 30, 31].

Given the complexity of perioperative care of PDAC and the intensive resources and support PDAC patients require, and increasing social isolation in society at large, we sought to investigate the impact of marital status on surgery and multimodality therapy in patients with PDAC using a large national cohort. We hypothesized *a priori* that married status, as a proxy for social support, would be correlated to higher rates of treatment attainment and reduced treatment delay.

## 2 Materials and Methods

### 2.1 Study Population

This study utilized the Surveillance, Epidemiology, and End Results‐17 (SEER‐17) incidence database to identify patients diagnosed with American Joint Committee on Cancer‐8th edition (AJCC‐8) stage I‐III PDAC from 2018 to 2022. Filtering for PDAC was conducted via International Classification of Diseases‐O‐3 histology and behavior codes. This study was exempt from institutional review board approval at the University of Pennsylvania given that it utilized deidentified data from a national database.

### 2.2 Patient‐, Treatment‐, and Disease‐Related Variables

Specific patient variables collected included age, sex (male, female), race (White, Black, other), marital status (married, widowed, divorced, single), household income, and rural‐urban designation. Disease‐related variables included AJCC‐8 stage. Treatment‐related variables collected included type of treatment received (surgery, systemic therapy, radiation therapy), time to surgery, and time to initial therapy (combined surgery or non‐surgery). Treatment delay was defined as time from diagnosis to initiation of first treatment of > 6 weeks.

### 2.3 Outcome Measures and Statistical Analyses

Specific outcome measures of interest included rates of surgery when recommended, rates of systemic/radiation therapy, time to treatment (TTT; surgery or non‐surgery), and treatment delay (surgery or non‐surgery).

The overall study population was presented using descriptive statistics, with medians with interquartile range (IQR) and/or means with standard deviation reported for continuous variables. Continuous variables were analyzed using Wilcoxon rank‐sum tests and unpaired t‐tests, while categorical variables were analyzed with Pearson's chi‐squared tests and Fisher's exact tests as appropriate.

The overall cohort was analyzed using univariable analysis stratified by marital status. Association of married status with sociodemographic factors was also assessed via multivariable logistic regression.

Multivariable logistic regression was used to assess odds of undergoing surgery or composite multimodality treatment; variables included in the model were marital status, sex, race, age, AJCC‐8 stage, household income, and rural‐urban residence status. Results were reported as adjusted odds ratios (aOR) with 95% confidence intervals (95% CI). An additional multivariable multinomial regression model was used to assess time to surgery and time to multimodality treatment, and included the following variables: marital status, sex, race, age, AJCC‐8 stage, household income, and rural‐urban residence status. Results were reported as difference in days with 95% CI.

Data on TTT was coded for only a portion of the overall cohort, thus analyses involving this variable were conducted as subset analysis of the overall cohort. Additional patients with unreported values were excluded from multivariable regressions as appropriate but were reported in the overall cohort descriptors (Table 1 and Table 2). The time to surgery was calculated directly for patients who underwent surgery. For calculations involving time to any treatment (surgery, systemic therapy, or radiation), the time to first treatment was utilized, whether it was surgery or systemic therapy/radiation. Treatment delay was calculated using time to any initial treatment (surgery, systemic therapy, or radiation). Results were reported as aORs with 95% CIs.

**Table 1:** Overall cohort description stratified by marital status.

Characteristic	Married or partnered (*N* = 14 280)	Divorced or separated (*N* = 2731)	Single/never‐married (*N* = 3665)	Widowed (*N* = 3864)	*p*
**Sex**					< 0.001
Female	5790 (40.55)	1636 (59.90)	1806 (49.28)	2971 (76.89)	
Male	8490 (59.45)	1095 (40.10)	1859 (50.72)	893 (23.11)	
**Race**					< 0.001
White	11 509 (80.68)	2125 (77.81)	2510 (68.49)	3070 (79.45)	
Black	1098 (7.69)	398 (14.57)	845 (23.06)	432 (11.18)	
Other	1630 (11.41)	194 (7.10)	284 (7.75)	342 (8.85)	
Unknown	43 (0.3)	14 (0.5)1	26 (0.71)	20 (0.52)	
**Age (years)**					< 0.001
< 65	4021 (28.16)	918 (33.61)	1602 (43.71)	271 (7.01)	
≥ 65	10 259 (71.84)	1813 (66.39)	2063 (56.29)	3593 (92.99)	
**AJCC Stage**					< 0.001
Stage I	4722 (33.07)	878 (32.15)	1142 (31.16)	1538 (39.8)	
Stage II	3414 (23.91)	663 (24.28)	804 (21.94)	732 (18.94)	
Stage III	6144 (43.03)	1190 (43.57)	1719 (36.90)	1594 (41.25)	
**Household Income**					< 0.001
Below $65 000	2048 (14.34)	459 (16.81)	562 (15.33)	673 (17.42)	
$65 000–$84 999	3782 (26.48)	748 (27.39)	971 (26.49)	1006 (26.04)	
Unreported	13 (0.09)	4 (0.15)	2 (0.05)	2 (0.05)	
**Rural‐Urban Designation**					0.001
Metropolitan Counties (population > 1 000 000)	8747 (61.25)	1611 (58.99)	2340 (63.85)	2300 (59.52)	
Metropolitan Counties (population 250 000–1 000 000)	2822 (19.76)	568 (20.80)	734 (20.03)	768 (19.88)	
Metropolitan Counties (population < 250 000)	1049 (7.35)	218 (7.98)	235 (6.41)	295 (7.63)	
Nonmetropolitan Counties (adjacent to metro counties)	1041 (7.29)	201 (7.36)	213 (5.81)	321 (8.31)	
Nonmetropolitan Counties (not adjacent to metro counties)	608 (4.26)	129 (4.72)	141 (3.85)	178 (4.61)	
Unreported	13 (0.09)	4 (0.15)	2 (0.05)	2 (0.05)	

*Note: p*‐values reflect 4‐way analyses between married, divorced, single, and widowed patients.

**Table 2:** Treatment characteristics stratified by marital status.

Characteristic	Married or partnered (*N* = 14 280)	Divorced or separated (*N* = 2731)	Single/never‐married (*N* = 3665)	Widowed (*N* = 3864)	*p*
**Cancer Directed Surgery (*n*, %)**					< 0.001
Surgery Recommended	6281 (43.98)	1094 (40.06)	1357 (37.03)	1145 (29.63)	
Surgery Performed	5650 (39.57)	944 (34.57)	1191 (32.50)	898 (23.24)	
Surgery Not Recommended	7809 (54.68)	1590 (58.22)	2302 (62.81)	2655 (68.71)	
Unreported	190 (1.33)	47 (1.72)	56 (1.53)	64 (0.88)	
**Systemic Therapy ‐ Surgery Sequence (*n*, %)**					< 0.001
No systemic therapy and/or surgical procedures	9179 (64.28)	1891 (0.04)	2660 (72.58)	3,182 (82.35)	
Systemic therapy before surgery	1500 (10.50)	251 (9.19)	320 (8.73)	199 (5.15)	
Systemic therapy both before and after surgery	1129 (7.91)	202 (7.40)	180 (4.91)	131 (3.39)	
Systemic therapy after surgery	2450 (17.16)	380 (13.91)	497 (13.56)	350 (9.06)	
Unreported	22 (0.15)	7 (0.26)	8 (0.22)	2 (0.05)	
**Radiation Therapy‐Surgery Sequence (*n*, %)**					< 0.001
No radiation and/or no surgery	13 019 (91.17)	2518 (92.20)	3417 (93.23)	3709 (95.99)	
Radiation before surgery	523 (3.66)	82 (3.00)	91 (2.48)	53 (0.03)	
Radiation before and after surgery	15 (0.11)	5 (0.18)	0 (0.00)	6 (0.16)	
Radiation after surgery	707(4.95)	124 (4.54)	155 (4.23)	94 (2.43)	
Unreported	16 (0.11)	2 (0.07)	2 (0.05)	2 (0.08)	
**Median Time to Surgery (days)**	26 (14–39)	29 (15–42)	28 (13–43)	30 (17–45)	< 0.001
**Median Time to Any Treatment (days)**	29 (19–43)	33 (21–48)	33 (19–50)	34 (22–50)	< 0.001
**Delayed Time to Treatment**	3019 (26.17)	651 (31.99)	875 (32.99)	788 (35.05)	< 0.001

*Note: p*‐values reflect 4‐way analyses between married, divorced, single, and widowed patients.

This study followed the STROBE guidelines for observational studies. An alpha level of 0.05 was applied to designate statistical significance. All analyses were conducted using Stata software version 17 (StataCorp).

## 3 Results

### 3.1 Patient Population of Clinicopathologic Characteristics

Of the eligible 24 540 patients that were diagnosed with stage I‐III PDAC, 14 280 (58.2%) were married or partnered, 2731 (9.7%) were divorced or separated, 3665 (14.9%) were single (never married), and 3864 (15.7%) were widowed (Table 1). 12 337 (48.5%) patients were male, 19 214 (75.5%) were White, and 17 728 (69.7%) were ≥ 65 years of age.

On univariable analysis, married patients diagnosed with PDAC were more likely to be male (59.5%, *p* < 0.001), White (80.7%, *p* < 0.001), ≥ 65 years old (71.8%, *p* < 0.001), and have a household income of ≥ $100,000 (31.5%, *p* < 0.001). Similarly on multivariable analysis, males (aOR: 2.45, 95% CI: 2.32–2.58, *p* < 0.001) and patients with income >$100 000 (aOR: 1.18, 95% CI: 1.06–1.32, *p* = 0.003) were more likely to be married, whereas Black patients (aOR: 0.45, 95% CI: 0.41–0.49, *p* < 0.001) were less likely to be married (Supporting Table 1). Detailed clinical and demographic characteristics stratified by each relationship status category are displayed in Table 1.

### 3.2 Treatment Characteristics

6 281 (44%) married patients were recommended to undergo surgery, compared to 1,094 (40%) divorced, 1357 (37%) single, and 1145 (30%) widowed patients (*p* < 0.001). Additionally, within patients that had surgery recommended, 90% of married patients had surgery performed; in comparison, this metric was 86% for divorced patients, 88% for single patients, and 78% for widowed patients (*p* < 0.001, Table 2). Likewise, rates of systemic therapy and radiation therapy were highest among married patients (Table 2).

Additionally, median time from diagnosis to surgery for those who underwent surgery as first treatment was 26 days (IQR: 14–39) for married patients, compared to 29 (IQR 15–42), 28 (IQR 13–43), and 30 (IQR 17–45) days for divorced, single, and widowed patients, respectively (*p* < 0.001, Table 2). This trend persisted when analyzed for “any treatment” as first treatment as well, which included surgery, systemic therapy, or radiation therapy; married patients received treatment at a median of 29 (IQR 19–43) days from diagnosis, compared to 33 (IQR 21–48), 33 (IQR 19–50), and 34 (IQR 22–50) days for divorced, single, and widowed patients respectively (*p* < 0.001, Table 2).

### 3.3 Multivariable Logistic Regression in Likelihood of Undergoing Surgery When Recommended

Analysis of likelihood of undergoing surgery when recommended was notable for decreased odds associated with divorced (aOR: 0.61, 95% CI: 0.46–0.77, *p* < 0.001), single (aOR: 0.60, 95% CI: 0.49 – 0.80, *p* < 0.001), and widowed (aOR: 0.37, 95% CI: 0.30–0.45, *p* < 0.001) patients when compared to married patients (Table 3). This same trend persisted when including systemic therapy and radiation as treatment modalities, with aORs of 0.75 (95% CI: 0.67–0.81), 0.62 (95% CI: 0.56–0.67), and 0.44 (95% CI: 0.39–0.47) for divorced, single, and widowed patients, respectively (*p* < 0.001 each).

**Table 3:** Regression analyses of likelihood to undergo surgery when recommended or composite multimodality treatment.

	Multimodality treatment			Surgery		
Factor	Odds ratio	*p*	95% CI	Odds ratio	*p*	95% CI
**Marital Status (compared to Married)**						
Divorced	0.75	< 0.001	0.67, 0.81	0.61	< 0.001	0.46, 0.77
Single	0.62	< 0.001	0.56, 0.67	0.60	< 0.001	0.49, 0.80
Widowed	0.44	< 0.001	0.39, 0.47	0.37	< 0.001	0.30, 0.45
**Sex (compared to Females)**						
Males	0.84	< 0.001	0.79, 0.89	0.85	0.059	0.74, 1.03
**Race (compared to White)**						
Black	0.75	< 0.001	0.69, 0.84	1.11	0.484	0.81, 1.45
Other	0.75	< 0.001	0.68, 0.84	0.70	0.008	0.55, 0.92
Unknown	1.10	0.701	0.67, 1.62	1.40	0.653	0.32, 5.72
**Age (compared to below 65 years old)**						
≥ 65 years	0.46	< 0.001	0.45, 0.51	0.30	< 0.001	0.25, 0.39
**Stage (compared to Stage I)**						
Stage II	3.09	< 0.001	3.17, 3.68	4.66	< 0.001	4.13, 6.57
Stage III	0.92	0.018	0.86, 0.99	1.40	< 0.001	1.22, 1.72
**Household Income (compared to < $65 000)**						
$65 000–$84 999	1.08	0.174	0.97, 1.20	1.00	0.981	0.85, 1.43
$85 000–$99 999	1.15	0.033	1.01, 1.28	1.21	0.244	0.94, 1.73
$100 000 +	1.32	< 0.001	1.18, 1.50	1.30	0.117	1.03, 1.94
**Rural‐Urban Designation (compared to Metropolitan Counties with Population** > **1 000 000)**						
Metropolitan Counties (population 250 000–1 000 000)	1.04	0.316	0.99, 1.15	0.75	0.011	0.61, 0.94
Metropolitan Counties (population < 250 000)	1.01	0.931	0.85, 1.20	0.75	0.107	0.52, 1.02
Nonmetropolitan Counties (adjacent to metro counties)	1.01	0.843	0.87, 1.14	0.60	0.004	0.44, 0.86
Nonmetropolitan Counties (not adjacent to metro counties)	1.02	0.854	0.87, 1.22	0.37	< 0.001	0.27, 0.55

Additional factors associated with decreased likelihood of undergoing surgery were age ≥ 65 years (aOR: 0.30, 95% CI: 0.25–0.39, *p* < 0.001), counties with population between 250,000 and 1 000 000 (vs. counties > 1 000 000 population, aOR: 0.75, 95% CI: 0.61–0.94, *p* = 0.011), nonmetropolitan counties adjacent to metropolitan areas (aOR: 0.60, 95% CI: 0.44–0.86, *p* = 0.004), nonmetropolitan counties not adjacent to nonmetropolitan areas (aOR: 0.37, 95% CI: 0.27–0.55, *p* < 0.001), and “Other” race (vs. White, aOR: 0.70, 95% CI: 0.55–0.92, *p* = 0.008). Male patients trended towards decreased odds of undergoing surgery (aOR: 0.85, 95% CI: 0.74–1.03, *p* = 0.059).

Compared to stage I, patients with stage II (aOR: 4.66, 95% CI: 4.13–6.57, *p* < 0.001) and stage III (aOR: 1.40, 95% CI: 1.22–1.72, *p* < 0.001) disease were more likely to undergo surgery.

### 3.4 Multivariable Analysis of Time to Surgery and Treatment Delay

Multivariable regression of time to surgery was notable for an increase of 4.5 (95% CI: 2.36–6.57), 4.0 (95% CI: 2.13–5.96), and 5.0 (95% CI: 2.81–7.20) days from diagnosis to surgery for divorced, single, and widowed patients compared to married patients, respectively (*p* < 0.001 each, Table 4). When including multimodality therapy, the same trend persisted, with an increase of 4.7 (95% CI: 3.13–6.29), 4.8 (95% CI: 3.38–6.25), and 5.5 (95% CI: 3.97–7.08) days for divorced, single, and widowed patients respectively (*p* < 0.001 each, Table 4).

**Table 4:** Regression analyses of time to first treatment, time to surgery, and likelihood of delayed treatment.

Factor	Time to first treatment			Time to surgery			Treatment delay (TTT > 6 weeks)		
	Coefficient (days)	*p*	95% CI	Coefficient (days)	*p*	95% CI	Odds Ratio	*p*	95% CI
**Marital Status (compared to Married)**									
Divorced	4.7	< 0.001	3.13, 6.29	4.5	< 0.001	2.36, 6.57	1.36	< 0.001	1.23, 1.54
Single	4.8	< 0.001	3.38, 6.25	4.0	< 0.001	2.13, 5.96	1.43	< 0.001	1.30, 1.57
Widowed	5.5	< 0.001	3.97, 7.08	5.0	< 0.001	2.81, 7.20	1.46	< 0.001	1.32, 1.62
**Sex (compared to Females)**									
Males	1.2	0.017	0.24, 2.22	1.0	0.150	0.35, 2.27	1.14	< 0.001	1.07, 1.22
**Race (compared to White)**									
Black	4.2	< 0.001	2.65, 5.82	2.7	0.018	0.46, 5.00	1.30	< 0.001	1.17, 1.44
Other	1.2	0.164	−0.44, 2.83	−1.0	0.380	−3.23, 1.23	1.08	0.184	0.97, 1.20
Unknown	−2.9	0.484	−10.86, 5.16	−0.6	0.902	−10.88, 9.59	0.74	0.316	0.42, 1.33
**Age (compared to** < **65 years)**									
≥ 65 years	3.6	< 0.001	0.24, 2.22	3.4	< 0.001	2.02, 4.74	1.38	< 0.001	1.28, 1.48
**Stage (compared to Stage I)**									
Stage II	−6.0	< 0.001	−7.88, −5.36	−1.0	0.197	−2.61, 0.54	0.67	< 0.001	0.62, 0.73
Stage III	−3.7	< 0.001	−4.84, −2.59	−0.3	0.699	−1.97, 1.32	0.79	< 0.001	0.74, 0.85
**Household Income (compared to <$65 000)**									
$65 000–$84 999	−0.9	0.339	−2.67, 0.90	−0.4	0.732	−2.85, 2.01	0.90	0.070	0.80, 1.01
$85 000–$99 999	−1.7	0.101	−3.62, 0.35	−2.5	0.069	−5.15, 0.20	0.89	0.095	0.78, 1.02
$100 000+	−2.0	0.047	−4.14, −0.09	−2.3	0.093	−5.07, 0.39	0.82	0.005	0.72, 0.94
**Rural‐Urban Designation (compared to Metropolitan Counties with Population** > **1 000 000)**									
Metropolitan Counties (population 250 000–1 000 000)	−0.7	0.289	−2.08, 0.54	−0.4	0.671	−2.09, 1.35	0.93	0.114	0.85, 1.02
Metropolitan Counties (population < 250 000)	0.0	0.984	−2.02, 2.23	−0.2	0.920	−3.10, 2.80	1.04	0.553	0.91, 1.20
Nonmetropolitan Counties (adjacent to metro counties)	−1.4	0.236	−3.61, 0.92	−1.0	0.537	−4.06, 2.11	0.91	0.245	0.78, 1.06
Nonmetropolitan Counties (not adjacent to metro counties)	0.3	0.827	−2.64, 3.11	0.9	0.654	−2.90, 4.61	1.10	0.315	0.91, 1.33

Proportion of patients experiencing treatment delay, defined as initiation of treatment > 6 weeks from diagnosis, was 26% for married patients versus 32%, 33%, and 35% for divorced, single, and widowed patients, respectively (*p* < 0.001, Table 2). On multivariable analysis, likelihood of treatment delay was notably higher for divorced (aOR: 1.36, 95% CI: 1.23–1.54, *p* < 0.001), single (aOR: 1.43, 95% CI: 1.30–1.57, *p* < 0.001), and widowed (aOR: 1.46, 95% CI: 1.32–1.62, *p* < 0.001) patients compared to married patients (Table 4).

## 4 Discussion

In this large national cohort analysis of patients with stage I‐III PDAC, we found that marital status was associated with increased rates of surgical treatment, faster time to surgery and multimodality therapy, and reduced rates of treatment delay. Conversely, patients that were divorced, single, or widowed exhibited worse treatment metrics compared to married individuals. These data suggest that marital status, as a proxy for social support, likely plays a multifactorial role in the treatment of PDAC.

Marital status may be associated with improved treatment metrics for multiple reasons. These potentially include motivation to initiate and adhere to treatment and more frequent interactions with the healthcare system in the form of screenings/follow‐up. Social support systems may include children, friends, communities, and organizations of which the patient is a member, for which marital status may serve as a proxy. It may also indicate greater access to tangible/financial resources and support in decision‐making regarding complex treatments [22, 23, 24, 25, 26, 27].

Importantly, although the absolute difference in median TTT between groups was modest (~4–6 days), this finding should be interpreted in the context of the broader treatment patterns observed. Specifically, non‐married patients were not only treated later on average but were also significantly more likely to experience clinically meaningful delays exceeding 6 weeks and less likely to undergo indicated treatment, both which have been closely linked with worse outcomes [14, 15, 32]. As such, the observed difference in TTT may represent an early signal of more substantial disparities in treatment access and delivery, rather than an isolated or clinically negligible delay.

These data are important in light of recent trends in marriage and social connectedness. Since 1949, when marriage rates peaked at 78.8%, rates have been steadily decreasing, reaching an all‐time low of 46.8% in 2022 [33], with some decline also correlated with the COVID‐19 pandemic [34]. For males, the average age of first marriage was 32 years old in 2025 in the USA, reflecting a 10‐year increase since the 1950s when the average age of marriage was 22.8 years old [35]. A similar trend is observed in females, with an average age of 30 years for first marriage versus 20.3 in 1950 [35]. Despite the absolute number of marriages and divorces decreasing, divorce rates for those 50 years and older have doubled since 1990 [33, 36].

The aforementioned trends in marital status correspond with broader trends of social isolation. Social isolation amongst adults ≥ 65 years is self‐reported to be as high as 24%, with unmarried status independently associated with increased isolation [31]. Trends in social isolation within the USA across decades point to adults ≥ 65 years, males, and Black individuals being at particularly high risk [29, 30]. On assessment of factors associated with married status in this current study, we found that race, age, and income were significantly associated. Indeed, we also found that in addition to social isolation based on marital status, adults ≥ 65 years and Black individuals were independently at higher risk for reduced treatment rates and delays in TTT (Table 4). Coinciding with this finding, widowed patients, who are more likely to be older in age, also demonstrated the lowest rates of treatment. The aforementioned findings highlight the multifactorial role of social determinants of health on both social isolation and treatment outcomes.

The rise of social isolation and incidence of PDAC will be increasingly pertinent to healthcare outcomes in the coming years. Specifically in the treatment of PDAC, which involves frequent pre‐ and postoperative interfacing with the healthcare system and necessitates complex multimodal approaches with high potential for complications [37], maximizing all avenues of treatment optimization is prudent. In terms of clinical practice implementation, our study highlights the need for healthcare systems to be aware of individuals at higher risk of social isolation presenting for cancer care, and in turn, mitigate these risks. Both medical and surgical oncology physicians and ancillary providers should be aware of these risks in order to optimize outcomes for PDAC patients in a holistic manner.

Specifically, it may be necessary to screen patients explicitly when they present for consultation for certain at‐risk criteria in their social history. Identification of higher‐risk groups such as divorced and widowed patients could be utilized to provide additional resources early on in the PDAC treatment process, including but not limited to psychosocial support, shared decision‐making, and practical/tangible support (i.e., financial, transportation, home healthcare, and patient navigation) [22, 23, 26, 38, 39]. These changes can be implemented within the electronic medical record systems already established at comprehensive cancer centers/networks.

Importantly, findings from our study also highlight potential provider‐level biases with regards to treatment recommendations. While surgery rates among non‐married patients were lower compared to married patients, the recommendation to undergo surgery itself was also significantly lower for non‐married patients to begin with. This disparity may further amplify reduced rates of treatment for more at‐risk social groups. Physicians should be particularly cognizant of recommending evidence‐based treatments in line with individual patient goals, ensuring that perceived ability to undergo therapy based on social determinants of health does not preclude treatment being recommended. Instead, individuals at risk for treatment failure should be readily identified and supported throughout the treatment continuum.

There are several limitations to this study, most notably those inherent to its retrospective design and analysis. Specifically, while we were able to control for many relevant clinicopathologic and demographic variables in our analysis, limited tumor‐specific data was available in the SEER‐17 registry, which may have affected our outcomes of interest. Furthermore, treatment‐specific information with regards to the type of therapy utilized lacks certain granularity (e.g., types of systemic therapy, number of cycles administered). In line with this, the specific reason for refusal of surgery or treatment are not provided within SEER‐17, which could add important context toward the interpretability of our findings. Additionally, time to surgery/treatment was not coded for a portion of patients, thus any analyses using that variable are only on a subset of the overall study cohort. Along with this, there are additional social factors not available within SEER‐17 that may also influence these metrics, such as broader categorization of social support networks. Lastly, while this analysis defines an area of PDAC treatment that is understudied and can potentially be addressed, future prospective and intervention‐based studies are needed to validate these findings.

## 5 Conclusion

In this retrospective national cohort analysis of patients with stage I‐III PDAC, we found that married patients were more likely to undergo surgery and multimodality therapy, and less likely to experience treatment delay, compared to divorced, single, and widowed patients. Comparatively, widowed patients displayed the worst treatment metrics and represent a particularly vulnerable patient population undergoing treatment for PDAC that may require additional social and tangible support in the perioperative period to improve rates of treatment attainment.

## Funding

The authors have nothing to report.

## Conflicts of Interest

The authors declare no conflicts of interest.

## Data Availability Statement

The data utilized in this study are available in the SEER database for authorized users.

## Synopsis

Married patients with pancreatic adenocarcinoma were more likely to undergo treatment, and less likely to experience treatment delay, compared to divorced, single, and widowed patients. Comparatively, widowed patients displayed the worst metrics and represent a particularly vulnerable patient population that may require additional support to improve rates of treatment attainment.

## Supporting information


Supporting File:
