# Supplementary material for: Marital Status Is Associated With Treatment Attainment in Pancreatic Adenocarcinoma
Source: J Surg Oncol. 2026 Apr 8;133(8):1050–8. doi: 10.1002/jso.70258 (PMC13353123; doi:10.1002/jso.70258)
Supplement: Supplementary file 1 — Supporting File: [file JSO-133-1050-s001.docx]

| **Supplemental Table 1:** Multivariable logistic regression of select sociodemographic factors associated with married status (vs. non-married). | | | |
| --- | --- | --- | --- |
| Factor | Odds Ratio | 95% CI | P-value |
| **Sex (compared to Females)** | | | |
| Males | 2.45 | 2.32, 2.58 | <0.001 |
| **Race (compared to White)** | | | |
| Black | 0.45 | 0.41, 0.49 | <0.001 |
| Other | 1.40 | 1.27, 1.53 | <0.001 |
| **Age (compared to Below 65 years old)** | | | |
| ≥ 65 years | 0.95 | 0.90, 1.01 | 0.118 |
| **Household Income (compared to <$65,000)** | | | |
| $100,000+ | 1.18 | 1.06, 1.32 | 0.003 |
